# Serum *p*-Cresyl Sulfate Is Independently Associated with Aortic Stiffness in Non-Dialysis Chronic Kidney Disease Patients

**DOI:** 10.3390/life15071116

**Published:** 2025-07-16

**Authors:** Yahn-Bor Chern, Ken Lee Chia, Chin-Hung Liu, Yu-Li Lin, Jen-Pi Tsai, Bang-Gee Hsu

**Affiliations:** 1 Division of Nephrology, Department of Internal Medicine, Yuan’s General Hospital, Kaohsiung 80249, Taiwan; 2 Department of Internal Medicine, Hualien Tzu Chi Hospital, Buddhist Tzu Chi Medical Foundation, Hualien 97004, Taiwan; 3 Graduate Institute of Clinical Pharmacy, School of Medicine, Tzu Chi University, Hualien 97004, Taiwan; 4 School of Pharmacy, Tzu Chi University, Hualien 97004, Taiwan; 5 Division of Nephrology, Hualien Tzu Chi Hospital, Buddhist Tzu Chi Medical Foundation, Hualien 97004, Taiwan; 6 School of Medicine, Tzu Chi University, Hualien 97004, Taiwan; 7 Division of Nephrology, Department of Internal Medicine, Dalin Tzu Chi Hospital, Buddhist Tzu Chi Medical Foundation, Chiayi 62247, Taiwan

**Keywords:** carotid–femoral pulse wave velocity, *p*-Cresyl sulfate, chronic kidney disease, aortic stiffness, biomarker

## Abstract

*p*-Cresyl sulfate (PCS), a gut-derived uremic toxin with proinflammatory and cytotoxic effects, has been implicated in cardiovascular injuries among patients with chronic kidney disease (CKD). Aortic stiffness (AS), assessed by carotid–femoral pulse wave velocity (cfPWV), is a recognized predictor of cardiovascular risk. This study investigated the association between serum PCS levels and AS in patients with nondialysis-dependent CKD. In total, 165 patients with nondialysis-dependent CKD were enrolled. Clinical data and fasting blood samples were collected. Arterial stiffness (AS) was assessed bilaterally by measuring carotid–femoral pulse wave velocity (cfPWV) on both the left and right sides. A value above 10 m/s was considered indicative of increased stiffness. Serum PCS levels were quantified using high-performance liquid chromatography–mass spectrometry. Fifty patients (30.3%) had AS. The AS group was significantly older and had higher diabetes prevalence, systolic blood pressure, fasting glucose, urinary protein-creatinine ratio, and PCS levels than the control group. In the multivariate analysis, both PCS (odds ratio [OR]: 1.097; 95% confidence interval [CI]: 1.024–1.175; *p* = 0.008) and age (OR: 1.057; 95% CI: 1.025–1.090; *p* < 0.001) were independently associated with AS. In conclusion, elevated serum PCS and older age were independently associated with AS. Thus, PCS is a potential early marker of vascular damage in CKD.

## 1. Introduction

Chronic kidney disease (CKD) is frequently associated with cardiovascular (CV) complications, which are a leading cause of morbidity and mortality in patients with this condition [1,2,3]. CV complications are also a primary cause of death in patients with nondialysis-dependent CKD (nondialysis CKD), exhibiting a mortality rate of 34.7% according to Navaneethan et al.’s report [1]. Both traditional risk factors (e.g., hypertension and diabetes mellitus [DM]) and CKD-specific risk factors (e.g., mineral bone disorder, anemia, fluid overload, and uremic toxins) underscore the intricate relationship between CKD and CV disease (CVD) [4,5]. Patients with CKD may present with different types of CVD, of which aortic stiffness (AS) is a significant predictor of kidney disease progression and all-cause and CV mortality [6,7,8,9]. By measuring the carotid–femoral pulse wave velocity (cfPWV), which is currently the gold standard for the noninvasive assessment of central aortic stiffening, clinicians can detect subclinical AS and reinforce risk stratifications in patients at high risk for CVD [10,11,12].

*p*-Cresyl sulfate (PCS), a protein-bound uremic toxin generated by the bacterial metabolism of tyrosine and phenylalanine in the intestines, accumulates with progressive renal function impairment [13]. PCS has been linked to endothelial dysfunction, oxidative stress, and vascular calcification regulation, as well as systemic inflammation—all crucial contributors to vascular injury and remodeling [14]. In vitro and in vivo studies have indicated that PCS interferes with endothelial nitric oxide (NO) production, stimulates reactive oxygen species (ROS) production, and activates proinflammatory pathways, including NF-κB signaling [15,16,17,18]. These phenomena can be closely related to the pathophysiological mechanisms involved in AS development and progression. Although increasing evidence has reported that PCS is associated with adverse CV outcomes, the relationship between PCS and arterial stiffness, especially measured by cfPWV in the nondialysis CKD population, remains unclear. Thus, this study aimed to clarify the association of serum PCS levels with AS presence as evaluated by cfPWV, to gain further insights into the possible involvement of PCS in CKD-related vascular dysfunction.

## 2. Materials and Methods

### 2.1. Participants

Between April 2022 and December 2022, patients with nondialysis CKD aged 18 years or older were recruited using consecutive sampling from the nephrology outpatient department of a medical center in Hualien, Taiwan. CKD was defined by two estimated glomerular filtration rate (eGFR) values ≤ 60 mL/min/1.73 m^2^ calculated using the Chronic Kidney Disease Epidemiology Collaboration equation [19] and blood-sampled more than 3 months apart. All patients received multidisciplinary CKD care, including dietary salt and protein restriction and nephrotoxic agent avoidance. The Research Ethics Committee of Hualien Tzu Chi Hospital, Buddhist Tzu Chi Medical Foundation, approved this study (IRB108-219-A). We excluded patients who were in a state of any medical illness, including malignancies, chronic inflammatory diseases, acute heart failure episodes, or chronic obstructive pulmonary disease, during blood sampling and those who declined to give informed consent for the study. The final participants provided written informed consent before the study began. According to the Eighth Joint National Committee criteria, systolic blood pressure (SBP) ≥ 140 mmHg and/or diastolic blood pressure (DBP) ≥ 90 mmHg or antihypertensive agent use during the previous 2 weeks indicated hypertension. Furthermore, DM was considered if the patients had a fasting plasma glucose level of 126 mg/dL or higher on more than two occasions or if they were taking antidiabetic agents.

The sample size was determined according to the general principles of an observational study on correlations, which cited that a sample size of no less than 100–150 subjects is sufficient to detect moderate effect sizes with desirable power [20]. A sample size of 85 would have 80% power to detect a correlation coefficient of 0.3 between serum PCS concentration and AS at the 5% alpha level. Additionally, to detect a correlation coefficient of 0.3 between serum PCS levels and AS, with an alpha level of 0.05 and a power of 90%, this study must include at least 113 patients. To assess whether the sample size was adequate, we conducted a post hoc power analysis based on the sample correlation coefficient between the log-transformed PCS and cfPWV values (*r* = 0.275). On the basis of a two-tailed alpha level of 0.05, the study’s sample size of 165 supports a power of approximately 93% in finding statistical significance according to the established procedures for alpha in correlation work [21]. Such an outcome suggested that the sample size was sufficient in detecting a moderate relationship between PCS concentrations and AS, with high reliability.

### 2.2. Anthropometric Analysis

The anthropometric parameters were collected in the morning after overnight fasting. We recorded each participant’s body weight and height to the nearest 0.5 kg and 0.5 cm, respectively. The body mass index (BMI) was calculated by dividing the body weight (kg) by the height-squared (m^2^) [22,23,24].

### 2.3. Biochemical Investigations

After the patients had fasted for at least 8 h, their blood samples (5 mL) were collected and immediately centrifuged at 3000× *g* for 10 min. The values of serum fasting glycemic levels, blood urea nitrogen (BUN), creatinine, total cholesterol, triglycerides (TG), total calcium, phosphorus, and low-density lipoprotein cholesterol (LDL-C) were obtained using an autoanalyzer (Siemens Advia 1800; Siemens Healthcare GmbH, Henkestr, Erlangen, Germany) [22,23,24]. Additionally, the urine protein-to-creatinine ratio (UPCR) was checked through a spot urine test.

### 2.4. Measurement of Serum PCS Levels by High-Performance Liquid Chromatography–Mass Spectrometry

This study employed a Waters e2695 high-performance liquid chromatography system with a mass spectrometer (ACQUITY QDa, Waters Corporation, Milford, MA, USA) [25]. The analytical column was a Phenomenex Luna^®^ C18 (2) (5 µ, 250 × 4.60 mm, 100 Å) under the following conditions: column temperature, 40 °C; flow, 0.8 mL/min; and 30 µL injection. The elution of the mobile phase followed a binary gradient. The initial amount (95% [A] water including 0.1% formic acid/5% [B] methanol including 0.1% formic acid) was held for 1 min, followed by linearly increasing the solvent B content from 5% to 70% over 12 min and holding it for 2 min. Solvent B was reduced to 50% in 1 min and maintained for 2 min for column equilibration.

Given that the pretreated samples were analyzed simultaneously in either positive or negative ionization modes (i.e., PCS) using electrospray ionization, the liquid chromatography–mass spectrometry (LC-MS) gradient conditions were optimized. The instrument parameters were as follows: desolvation temperature, 600 °C; capillary voltage, 0.8 kV; sample cone voltage, 15.0 V; and the mass spectrometer working under full scan mode from 50 to 450 *m*/*z* (positive ion mode) and 100 to 350 *m*/*z* (negative ion mode). The specific masses of each compound (PCs: 187.0 *m*/*z*) were monitored using the single-ion recording mode. Data were acquired and processed using the Empower^®^ 3.0 software (Waters Corporation, Arapahoe, CO, USA). Approximately, PCS was retained for 16.56 min. Endogenous compounds were also determined. Moreover, the peak areas were measured and compared with respect to a calibration curve prepared from standard solutions. All *r^2^* values (linearity) were higher than 0.995. For the single-ion analysis, the single-ion recording mode of the LC-MS was used.

### 2.5. Measurements of Blood Pressure (BP) and cfPWV

After blood sampling, the patients rested for 10 min in the supine position. Morning BP was measured using an upper-arm automatic oscillometric device by the trained staff, and SBP and DBP at the right brachial artery were measured thrice with a 5 min interval and then averaged. AS was assessed by cfPWV using a cuff-based volumetric displacement device (SphygmoCor XCEL, AtCor Medical, Sydney, NSW, Australia) [26]. Before measurement, the participants were required to be at rest for at least 10 min in the morning while in the supine position in a quiet room with a controlled room temperature. Thereafter, the cuff of the XCEL device was positioned on the left upper arm, and the brachial SBP and DBP were measured automatically by the device with standard oscillometric measurements; then, the cuff was immediately reinflated to a sub-DBP level. Next, an upper-thigh cuff for femoral artery tonometry was replaced by volume-displacement waveforms through the XCEL system to measure cfPWV. Meanwhile, the carotid pulse was measured using tonometry. In an expert consensus guidance, a cfPWV > 10 m/s suggests AS [27].

### 2.6. Statistical Analysis

The normal distribution of the parameters was tested using the Kolmogorov–Smirnov test. Quantitative variables with normal and nonnormal distributions are expressed as mean ± standard deviation and median (interquartile range [IQR]), respectively, and they were compared between groups by using the two-tailed Student’s independent *t*-test and Mann–Whitney *U* test. For categorical variables, which are presented as numbers with percentages, the χ^2^ test was used for comparison. Furthermore, significant variables associated with AS in the univariate analyses were further analyzed using multivariate logistic regression. Given that triglyceride, fasting glucose, urine protein-creatinine ratio (UPCR), and PCS were non-normally distributed, base 10 logarithmic transformations were conducted to normalize them. Variables that were statistically associated with cfPWV values in patients with CKD were examined for independence by linear regression analysis and reconfirmed by multivariate forward stepwise regression analysis. The receiver operating characteristic (ROC) was calculated for the area under the curve (AUC) and cutoff value of serum PCS concentrations in predicting AS in patients with CKD (MedCalc Software Ltd., version 22.019, Ostend, Belgium). The relationship of the serum PCS levels with the variables was assessed using Spearman’s correlation coefficient analysis. All statistical data were analyzed using SPSS software for Windows (version 19.0; SPSS, Chicago, IL, USA) and R version 4.3.2 (R Foundation for Statistical Computing, Vienna, Austria). A *p*-value below 0.05 was considered statistically significant.

## 3. Results

Table 1 lists the basic characteristics of the 165 patients with CKD (115 in the control group and 50 in the AS group). The AS group had a considerably higher DM rate (*p* = 0.036), was significantly older (*p* < 0.001), and had more elevated SBP (*p* = 0.016), fasting glucose levels (*p* = 0.008), spot UPCR (*p* = 0.048), and serum PCS concentrations (*p* = 0.001) than the control group. The sex ratio, glomerulonephritis prevalence, hypertension, antihypertensive agent use, and CKD stage did not significantly differ between the two groups.

**Figure 1:**
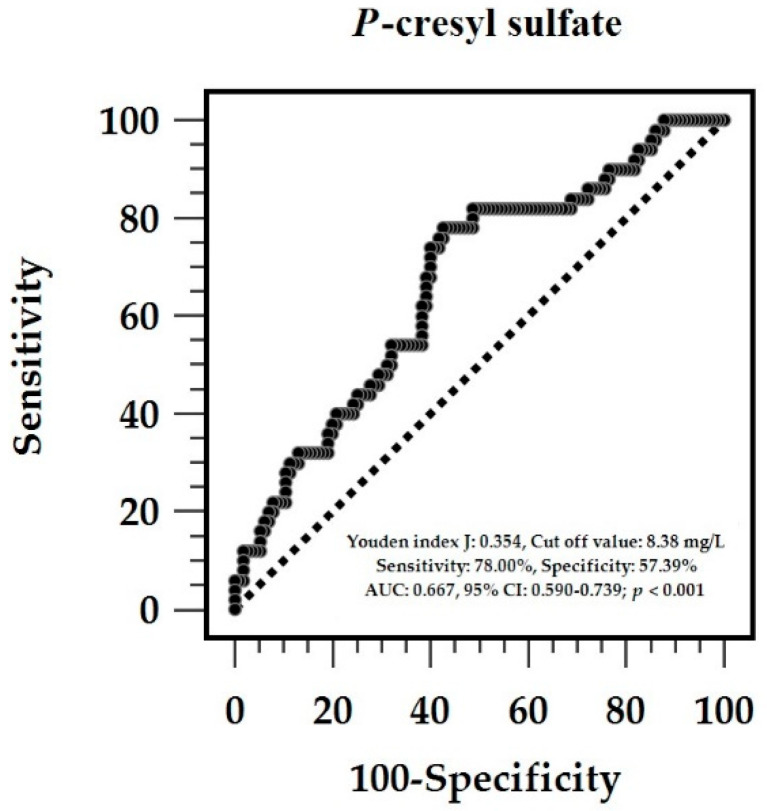
The area under the receiver operating characteristic (ROC) curve signifies the diagnostic efficacy of serum total *p*-Cresyl sulfate concentrations regarding the diagnosis of aortic stiffness in 165 individuals with chronic kidney disease. The solid line with dots is the ROC curve itself, which plots sensitivity (true positive rate) on the y-axis against 1 – specificity (false positive rate) on the x-axis. Each point on this curve corresponds to a different threshold of *p*-Cresyl sulfate concentration used to classify test results. The diagonal dashed line serves as a reference or “line of no discrimination,” representing a test with no diagnostic value (equivalent to random guessing).

After adjusting for factors significantly associated with AS (DM, age, SBP, fasting glucose, UPCR, and PCS), the multivariate logistic regression analysis showed that the serum PCS level (*p* = 0.008) and age (*p* < 0.001) were independent determinants of AS in patients with CKD (Table 2). The ROC curve of the PCS predicting AS had an AUC of 0.667 (*p* = 0.0002) (Figure 1). Based on 2000 stratified bootstrap replicates, the 95% confidence interval was 0.570–0.753, indicating moderate discriminatory ability. Moreover, the best PCS cutoff point for predicting AS was 8.39 mg/L, and the sensitivity, specificity, positive predictive value, and negative predictive value were 78.00%, 57.39%, 44.31%, and 85.72%, respectively. Considering age, SBP, and UPCR—three variables significantly associated with PWV in the multivariate regression models—we constructed joint models to assess predictive improvement. After adding age to PCS, the AUC significantly increased to 0.740 (95% CI: 0.654–0.818, based on 2000 stratified bootstrap replicates; DeLong test, *p* = 0.045), indicating improved discrimination. Further inclusion of SBP and UPCR did not lead to a statistically significant additional increase in AUC (0.753; 95% CI: 0.671–0.829, based on 2000 stratified bootstrap replicates; DeLong test, *p* = 0.302).

Table 3 presents the correlations between cfPWV and clinical variables in the CKD cohort. In simple linear regression analyses, DM (*p* = 0.043), age (*p* < 0.001), SBP (*p* < 0.001), log-transformed glucose (log-glucose; *p* = 0.033), log-UPCR (*p* = 0.014), and log-PCS (*p* < 0.001) were significantly correlated with cfPWV. In the multivariate stepwise linear regression analysis, all, except log-glucose, remained independently and positively associated with cfPWV values.

Table 4 summarizes the results of the Spearman’s correlation analysis between the levels of log-PCS and clinical features. Log-PCS levels correlated positively with SBP (*p* = 0.001), log-BUN (*p* = 0.035), log-creatinine (*p* = 0.007), and log-UPCR (*p* = 0.032) but negatively with eGFR (*p* = 0.029).

## 4. Discussion

In our study of patients with nondialysis CKD, the increased levels of serum PCS were significantly related to AS, which was reflected by cfPWV. PCS, as well as age, was found to be associated with AS in the multivariate analysis, adjusting for other potentially confounding risk factors. Additionally, the ROC curve analysis revealed that PCS could modestly distinguish AS (optimal cutoff value, 8.39 mg/L). A multivariate linear regression analysis was conducted to further investigate the clinical factors associated with the increase in cfPWV value. Results showed that older age, higher SBP, log-PCS, and log-UPCR contributed to increased cfPWV measurement. In the correlation analysis, higher PCS levels correlated with renal function markers, SBP, and cfPWV measurements. Therefore, PCS may be implicated in increased stiffness of the central arteries in patients with CKD, independent of the classic CVD risk factors, and may be a potential biomarker of early vascular injury in these patients.

The pathophysiologic mechanisms that explain the discriminative value of age, SBP, and UPCR to predict AS presence or the increased cfPWV values in patients with CKD are multiple and interrelated. First, extensive evidence has shown that AS is strongly related to aging because the arterial wall undergoes structural and functional changes, such as extracellular matrix remodeling and collagen accumulation, with aging [28,29,30], resulting in a gradual loss of arterial compliance and increased stiffness occur. Second, BP is a two-way issue in relation to AS presence. High SBP induces a mechanical stress on the arterial wall, leading to AS development by endothelial dysfunction, inflammation, and vascular smooth muscle cell hypertrophy [31,32]. Conversely, these stiffened arteries could induce BP elevations through higher left ventricular afterload, renal microvasculature overload, and small artery remodeling [31,32,33]. Lastly, a number of studies have consistently demonstrated an independent and powerful association between increasing proteinuria and arterial stiffness across different study populations, regardless of the nature of proteinuria (albuminuria or nonalbuminuria) [34,35,36,37,38]. Although the precise mechanisms are still poorly understood, proteinuria is closely interrelated with AS through multiple mechanisms, such as endothelial dysfunction, inflammation, and kidney injury [39,40,41,42]. Moreover, urinary protein excretion is an indicator of damage in the renal tubule and/or glomerulus and may be associated with increased inflammation, oxidative stress, and arterial remodeling in CKD [43]. Thus, the results regarding the correlations of old age, high SBP, and more proteinuria with AS/cfPWV in this study are consistent with previous knowledge, thereby reinforcing the validity and reliability of our findings.

Our study highlights the potential role of PCS in the occurrence of AS in patients with nondialysis CKD. PCS is a gut-derived protein-bound uremic toxin that exerts deleterious effects on arteries by promoting oxidative stress, inflammation, vascular calcification, and endothelial dysfunction, which are key pathophysiological processes contributing to arterial stiffening [44,45,46]. Several mechanisms can explain the positive correlations between PCS levels and renal dysfunction markers, including elevated BUN and creatinine, lower eGFR, higher proteinuria, and increased SBP. First, impaired renal clearance in CKD leads to PCS accumulation in the circulation, given that PCS is poorly removed by glomerular filtration and even less efficiently eliminated by tubular secretion [47,48]. Thus, elevated BUN and creatinine levels, which reflect reduced kidney excretory function, parallel PCS accumulation. Second, higher PCS levels are associated with increased proteinuria, possibly because of PCS-induced tubular toxicity, epithelial–mesenchymal transition, and glomerular barrier integrity disruption [49]. Third, the association between higher PCS levels and elevated SBP may be partially explained by PCS-mediated vascular remodeling, calcification, and arterial stiffness, which can all lead to increased peripheral resistance and hypertension [23,46]. PCS also activates the NADPH oxidase and NF-κB pathways, contributing to vascular inflammation and hypertrophy of vascular smooth muscle cells, further exacerbating hypertension [15,50]. Taken together, PCS may not only serve as a biomarker reflecting renal dysfunction but also actively participate in the pathogenesis of vascular injury and arterial stiffness. Clinically, the demonstrated association between PCS levels and AS strengthens the idea that PCS concentration monitoring could provide additional insights into CV risk stratification in patients with CKD. Interventions such as gut microbiota modulation (i.e., prebiotics, AST-120 adsorbent) and targeted toxin clearance can reduce PCS burden, thereby potentially attenuating AS progression [13,51]. Combining these strategies with rigorous BP control and RAAS inhibition could synergistically preserve vascular compliance, enabling the integrated management of uremic toxins and traditional risk factors in CKD care.

However, this study has several limitations that should be considered. First, it is a cross-sectional study; hence, a causal relationship between the high PCS levels and AS occurrence cannot be confirmed. Although we adjusted the regression results with many confounders, we could not completely exclude the probability of residual confounding by unmeasured factors, such as dietary behaviors, gut microbiota profiles, and the levels of inflammatory cytokines and oxidative stress markers. Second, physical activity, smoking history, and dietary patterns, which were associated with gut health and socioeconomic status, were not systematically evaluated as potential determinants of arterial stiffness, in addition to the classic risk factors for CVD and renal parameters. Third, PCS was measured as total instead of free and protein-bound PCS, which may have diverse biological activities and vascular effects. Fourth, this study examined patients with nondialysis CKD in a single center; thus, the findings might not be generalizable to wider CKD populations, especially those with dialysis treatment and from other races. Lastly, cfPWV, the gold standard for the noninvasive assessment of arterial stiffness, might still be affected by acute hemodynamic alterations during measurement. Such limitations underscore the necessity for additional longitudinal studies involving more numerous and diverse participants, more significant adjustment for confounders, and an exploration of the mechanisms through which our results may be confirmed.

## 5. Conclusions

This study showed that increased serum PCS was independently associated with the presence of AS, which can be reflected by cfPWV, in patients with nondialysis CKD. After controlling for potential confounding clinical factors, we found that PCS acted as an independent determinant of AS and was related to renal impairment markers and elevated SBP. Therefore, PCS may be a valuable biomarker for early vascular damage in high-risk CKD cases. Our findings have clinical relevance. Identifying PCS as a risk-modulating factor in AS adds to the increasing recognition of uremic toxins in CVD risk stratification among patients with CKD. By monitoring PCS levels, clinicians can conduct a more comprehensive assessment of vascular health and provide early interventions to ameliorate CV abnormalities in this high-risk population.

## Figures and Tables

**Table 1:** Clinical characteristics of the cohort comprising 165 patients diagnosed with chronic kidney disease, categorized based on the presence or absence of aortic stiffness.

Characteristics	All Patients (*n* = 165)	Control Group (*n* = 115)	Aortic Stiffness Group (*n* = 50)	*p*-Value
Age (years)	65.71 ± 13.98	63.07 ± 13.87	71.78 ± 12.35	<0.001 *
Body mass index (kg/m^2^)	26.01 ± 4.65	25.63 ± 4.73	26.90 ± 4.38	0.108
cfPWV (m/s)	9.26 ± 2.94	7.74 ± 1.45	12.75 ± 2.49	<0.001 *
SBP (mmHg)	147.27 ± 24.45	144.24 ± 22.46	154.22 ± 27.50	0.016 *
DBP (mmHg)	83.88 ± 13.78	83.26 ± 12.86	85.30 ± 15.75	0.384
Total cholesterol (mg/dL)	159.10 ± 40.59	156.56 ± 40.94	164.96 ± 39.56	0.223
Triglyceride (mg/dL)	119.0 (90.00–165.00)	116.00 (87.00–162.00)	133.50 (97.00–178.50)	0.119
LDL-C (mg/dL)	87.01 ± 33.72	84.94 ± 34.41	91.78 ± 31.92	0.232
Fasting glucose (mg/dL)	110.00 (98.50–139.50)	108.00 (97.00–132.00)	122.00 (100.75–166.00)	0.008 *
Blood urea nitrogen (mg/dL)	29.00 (22.00–43.50)	28.00 (22.00–44.00)	32.00 (22.75–43.25)	0.631
Creatinine (mg/dL)	1.70 (1.30–2.55)	1.70 (1.20–2.60)	1.80 (1.40–2.43)	0.419
eGFR (mL/min)	38.76 ± 24.96	40.13 ± 25.66	35.56 ± 23.18	0.279
Spot UPCR (g/g)	0.53 (0.19–1.20)	0.45 (0.16–1.08)	0.65 (0.24–1.95)	0.048 *
Total *p*-Cresyl sulfate (1 mg/L)	8.64 (6.25–12.63)	7.68 (6.08–11.63)	10.45 (8.53–15.34)	0.001 *
Female, n (%)	77 (46.7)	56 (48.7)	21 (42.0)	0.428
Diabetes mellitus, n (%)	69 (41.8)	42 (36.5)	27 (54.0)	0.036 *
Hypertension, n (%)	134 (81.2)	93 (80.9)	41 (82.0)	0.864
Glomerulonephritis, n (%)	41 (24.8)	31 (27.0)	10 (20.0)	0.342
ARB use, n (%)	103 (62.4)	75 (65.2)	28 (56.0)	0.261
β-blocker use, n (%)	46 (27.9)	32 (27.8)	14 (28.0)	0.982
CCB use, n (%)	68 (41.2)	45 (39.1)	23 (46.0)	0.410
Statin use, n (%)	80 (48.5)	54 (47.0)	26 (52.0)	0.551
Fibrate use, n (%)	47 (28.5)	30 (26.1)	17 (34.0)	0.301
CKD stage 1–2	26 (15.8)	20 (17.4)	6 (12.0)	0.775
CKD stage 3	65 (39.4)	46 (40.0)	19 (38.0)	
CKD stage 4	44 (26.7)	29 (25.2)	15 (30.0)	
CKD stage 5	30 (18.2)	20 (17.4)	10 (20.0)	

Variables for continuous data are presented as mean ± standard deviation, and the significance is tested by Student’s *t*-test; those not normally distributed are presented as median and interquartile range, and analysis was performed by the Mann–Whitney U test; values are expressed as number (%) and analyzed using the chi-square test. Abbreviations: cfPWV, carotid–femoral pulse wave velocity; SBP, systolic blood pressure; DBP, diastolic blood pressure; LDL-C, low-density lipoprotein cholesterol; eGFR, estimated glomerular filtration rate; UPCR, urine protein-to-creatinine ratio; ARB, angiotensin-receptor blocker; CCB, calcium-channel blocker; CKD, chronic kidney disease. * *p* < 0.05 was considered statistically significant.

**Table 2:** Multivariate logistic regression analysis assessing the factors associated with aortic stiffness in a cohort of 165 individuals diagnosed with chronic kidney disease.

Clinical Variables	Odds Ratio	95% Confidence Interval	*p*-Value
Total *p*-Cresyl sulfate, 1 mg/L	1.097	1.024–1.175	0.008 *
Age, 1 year	1.057	1.025–1.090	<0.001 *
Systolic blood pressure, 1 mmHg	1.006	0.989–1.023	0.477
Diabetes mellitus, present	1.275	0.487–3.366	0.624
Fasting glucose, 1 mg/dL	1.006	0.996–1.017	0.214
Spot UPCR, 1 g/g	1.108	0.871–1.410	0.404

Data analyses were performed using multivariate logistic regression analysis (the adjusted factors: diabetes mellitus, age, systolic blood pressure, fasting glucose, UPCR, and total *p*-Cresyl sulfate). Abbreviation: UPCR, urine protein-to-creatinine ratio. * *p* < 0.05 was considered statistically significant.

**Table 3:** Correlation between carotid–femoral pulse wave velocity measurements and clinical parameters among the 165 chronic kidney disease patients.

Clinical Variables	Carotid–Femoral Pulse Wave Velocity (m/s)				
	Univariate Regression		Multivariate Regression		
	*r*	*p*-Value	β	Adjusted R^2^ Change	*p*-Value
Age (years)	0.328	<0.001 *	0.311	0.102	<0.001 *
Body mass index (kg/m^2^)	0.107	0.170	–	–	–
SBP (mmHg)	0.282	<0.001 *	0.164	0.058	0.001 *
DBP (mmHg)	0.126	0.106	–	–	–
Total cholesterol (mg/dL)	0.021	0.793	–	–	–
Log-Triglyceride (mg/dL)	0.025	0.754	–	–	–
LDL-C (mg/dL)	0.023	0.772	–	–	–
Log-Glucose (mg/dL)	0.166	0.033 *	–	–	–
Log-BUN (mg/dL)	0.084	0.283	–	–	–
Log-Creatinine (mg/dL)	0.142	0.068	–	–	–
eGFR (mL/min)	−0.150	0.054	–	–	–
Log-UPCR (g/g)	0.191	0.014 *	0.148	0.015	0.046 *
Log-PCS (mg/L)	0.275	<0.001 *	0.181	0.032	0.007 *

Triglyceride, glucose, BUN, creatinine, UPCR and total PCS levels had a skewed distribution and were log-transformed prior to data analysis. Univariate linear regression analyses or multivariate stepwise linear regression analyses were used for data analysis (adjusted factors and covariates were diabetes mellitus, age, SBP, log-Glucose, log-UPCR, and log-PCS). Abbreviations: SBP, systolic blood pressure; DBP, diastolic blood pressure; LDL-C, low-density lipoprotein cholesterol; BUN, blood urea nitrogen; eGFR, estimated glomerular filtration rate; UPCR, urine protein-to-creatinine ratio; PCS, *p*-Cresyl sulfate. * *p* < 0.05 was considered statistically significant. The en dash in these cells indicates non-applicable values because they were not statistically significant in univariate analysis.

**Table 4:** Correction between log-transformed serum total *p*-Cresyl sulfate levels and clinical variables.

Variables	Spearman’s Correlation Coefficient	*p*-Value
Age (years)	0.087	0.269
Body mass index (kg/m^2^)	0.079	0.313
SBP (mmHg)	0.261	0.001 *
DBP (mmHg)	0.120	0.124
Total cholesterol (mg/dL)	−0.031	0.689
Log-Triglyceride (mg/dL)	0.009	0.910
LDL-C (mg/dL)	0.068	0.383
Log-Glucose (mg/dL)	0.135	0.083
Log-BUN (mg/dL)	0.164	0.035 *
Log-Creatinine (mg/dL)	0.209	0.007 *
eGFR (mL/min)	−0.170	0.029 *
Log-UPCR	0.167	0.032 *
Carotid–femoral PWV (m/s)	0.275	<0.001 *

Triglyceride, glucose, BUN, creatinine, UPCR, and *p*-Cresyl sulfate variables exhibited a skewed distribution and were log-transformed for analysis. Abbreviations: SBP, systolic blood pressure; DBP, diastolic blood pressure; LDL-C, low-density lipoprotein cholesterol; BUN, blood urea nitrogen; eGFR, estimated glomerular filtration rate; UPCR, ratio of urine protein to creatinine; PWV, pulse wave velocity. * *p* < 0.05 was considered statistically significant (2-tailed).

## Data Availability

The data presented in this study are available on request from the corresponding author. The data are not publicly available due to privacy and ethical restrictions involving patient confidentiality.

## Abbreviations

The following abbreviations are used in this manuscript:

ARIC	Atherosclerosis Risk in Communities
AS	Aortic stiffness
AUC	Area under the curve
BMI	Body mass index
BP	Blood pressure
BUN	Blood urea nitrogen
CKD	Chronic kidney disease
CVD	CV disease
DBP	Diastolic blood pressure
DM	Diabetes mellitus
PCS	*p*-Cresyl sulfate
ROC	Receiver operating characteristic
ROS	Reactive oxygen species
SBP	Systolic blood pressure
UPCR	Urine protein-to-creatinine ratio
